# The Role of Dementia Champions Across Health and Social Care Settings: Identifying Mechanisms of Action Using a Theory of Change Approach

**DOI:** 10.1177/14713012251410977

**Published:** 2026-01-23

**Authors:** Monica Leverton, Tiffeny James, Kritika Samsi, Christina Newton, Jill Manthorpe

**Affiliations:** 1 Health & Social Care Workforce Research Unit, King’s College London, London, UK; 2 Independent Researcher, UK

**Keywords:** dementia, dementia champions, health and social care, care workforce, theory of change

## Abstract

As the global prevalence of dementia continues to rise, expanding and upskilling the health and social care workforce is a top priority. To address this need, some health and social care services have implemented Dementia Champions (DCs). DCs are typically existing staff who are recognised and developed as dementia specialists to advocate for the needs of people affected by dementia within their service. However, evidence around the DC role across health and social care is limited. In this narrative review, we aimed to understand and define the DC role by exploring who becomes a DC; where they work; what their tasks and responsibilities are; and what is needed for the role to be successful. For the latter, we aimed to develop a Theory of Change framework for implementing, embedding, and maintaining the DC role in health and social care settings. A systematic search of the literature identified DCs working predominantly in hospitals and care home settings with a notable absence of the DC role within homecare services. Tasks and responsibilities of the DC role varied between settings and services. Findings highlighted that to be successful, the role needs clear definitions and boundaries; organisational and managerial support and buy-in (including establishing protected time for the role); and ongoing professional development. While DCs are implemented in pockets of the health and social care workforce, the role is not well established. Further research is needed to assess the impact of DCs on workforce effectiveness and staff well-being, patient and family carer outcomes, and cost-effectiveness in diverse health and social care settings.

## Introduction

### Dementia and the Care Workforce

Expanding and building capacity of the health and social care workforce in dementia are global priorities (Prince et al., 2016). Over 55 million people live with dementia worldwide, with 10-million new diagnoses annually (World Health Organisation, 2023). Increasing demand for dementia care requires considerable growth in numbers of both unpaid family carers and the paid care workforce (Gilster et al., 2018; Skills for Care, 2023).

There is global concern about the workforce’s preparedness to deliver high-quality dementia services (American Public Health Association, 2020). To address this, expansion and upskilling of the workforce are key, focusing on recruitment, retention, training, and funding to support skilled care. Educational and training interventions can build workforce capacity. Knowledge sharing models such as champion roles or mentors are amongst successful components of staff training programmes (Surr et al., 2017, 2020). However, evidence on the effectiveness and impact of such interventions is limited.

### What is ‘Championing’ Work?

A champion is someone who enthusiastically supports, defends, or fights for a person, belief, or cause. In a health and social care context, champions may advocate for an initiative or for the needs of a specific group of people in the service they work in. Champions are often members of staff who offer or are asked by their managers to take on the role in addition to their existing role. The expectations of the role can vary greatly between services, but may include educating and training staff; developing information leaflets and posters; building relationships with stakeholders; being visibly identified with the initiative/cause; and troubleshooting problems with implementation of or engagement with the initiative/cause (Miech et al., 2018). The term has been used in Australia, Canada, Japan, the UK, and USA (Miech et al., 2018), with championing roles in various capacities including: champions of pain (Tran et al., 2024); diabetes (Maryniuk et al., 2013); cultural competence (Handtke et al., 2019); and organisational champions (Hendy & Barlow, 2012). In 2004, the Department of Health in England (2004) released a resource toolkit for Older People’s Champions, defining champions as individuals with a desire to improve older people’s services and advocate for their interests. A survey of 209 Older People’s Champions in health and care found their work focused on communication, leadership, networking, and service improvement and evaluation (Manthorpe, 2004).

There is growing evidence for the potential effectiveness of championing roles in care settings (Bunce et al., 2020), however, evidence around longer-term effectiveness on staff wellbeing and patient care quality is sparse. There is some suggestion that while championing roles are beneficial to begin with, their effectiveness may diminish overtime (Hendy & Barlow, 2012). Championing is widely considered an approach to recognise valued staff members with greater experience, skill, and passion in a particular area (Edge et al., 2021). However, drawbacks to the role include lack of role clarity and expectations; limited information around how to prepare champions for the role; and unclear mechanisms for how champions can effectively enact change or have influence within services (Manthorpe, 2004; Miech et al., 2018).

### Dementia Champions (DCs)

“Dementia Champions” (DCs) have been implemented in some health and social care services in the UK and internationally to develop staff as dementia specialists. In Scotland, for example, more than 1000 health and care professionals working in hospitals have been trained as DCs through a pioneering programme developed by the University of West Scotland (Jack-Waugh et al., 2017). The programme aims develop champions as change agents for improving the experiences, care, treatment, and outcomes for people living with dementia and their families in hospitals in Scotland. Knowledge around the role of DCs in other countries is lacking.

In preparation for this review, we searched job websites (LinkedIn, Indeed, Guardian jobs, NHS jobs) between 1^st^ September – 1^st^ December 2022 for Dementia Champion roles in England (Turner et al., 2020). An initial search using the term “Dementia Champion” returned no results, so we broadened it to “Dementia Specialist”. Over the three-month period, we identified 13 job descriptions with the title “Dementia Champion” (n = 2) or ‘Dementia Specialist’ (n = 11). Seven roles were advertised in social care (homecare and residential care/nursing homes); three in healthcare (hospital and community settings); and three across both sectors. Tasks and responsibilities included: leadership; service evaluation; undertaking and/or delivering dementia training; providing dementia specialist support; staff supervision and development; and multidisciplinary working. Whilst service improvement (monitoring, evaluation, assessment) was mentioned in six of the 13 job descriptions, there was a general lack of consistency in the tasks and responsibilities across of a DC across settings suggesting that the DC role is not well defined within health and social care.

This article describes the first phase of a four-phase National Institute for Health and Care Research (NIHR) School for Social Care Research funded programme entitled ‘Developing the role of Dementia Champions in the homecare sector’ (the ‘DemChamp’ study). In this article, we aimed to define the DC role and understand how it is currently implemented in practice in the UK and internationally by identifying: who becomes a DC; where they work; what their tasks and responsibilities are; and what is needed for the role to work in practice i.e. what are the mechanisms of action required for the DC role to be implemented, embedded, and maintained within health and social care services.

## Methods

### Theoretical Framework

We used a Theory of Change (ToC) approach to conceptualise the DC role, drawing on the principles of realist methodology to establish what works for whom, in what settings, and how (Schäl, 1998). ToC is used to understand how and why an initiative works. For the DC role, this means understanding the tasks and responsibilities of a DC (the “activities” or “inputs”) as well as the mechanisms of action required for the role to be implemented (short-term mechanisms), embedded (medium-term), and maintained (long-term). ToCs can enhance stakeholder engagement to improve and refine an intervention in context, and provide a comprehensive map of how long-term outcomes can be achieved to reach the desired impact (De Silva et al., 2014).

### Design

We conducted a narrative review to identify and synthesise available data relevant to the research aim and objectives. A narrative review allowed us to widely explore the role of a DC as a concept across various data sources, which may have been restricted by other forms of review (Ferrari, 2015). We followed guidance by Ferrari (2015) for narrative reviews which recommends using distinctive search terms; setting inclusion/exclusion criteria to focus the relevance of information; and searching for a variety of information sources via database and manual searching until reaching saturation.

### Search

We searched Pubmed and CINAHL databases, as well as the King’s College London online library in August 2022 to identify national and international literature, grey literature, organisational and policy documents, news articles, and blogs. We hand-searched relevant sources, guided by the knowledge of our study advisory groups, adding identified texts up until February 2023. As this review aimed to define the DC role and understand how it is currently operationalised in practice, we searched only for the term “Dementia Champion”, using a systematic approach to add clarity and reduce bias (Ferrari, 2015; Yuan & Hunt, 2009).

### Procedure

We assessed all titles, abstracts, and main texts against our eligibility criteria (Table 1). We included texts published after 2010 which began the acceleration of championing work (Miech et al., 2018), particularly in the UK, following the Prime Minister’s Challenge on Dementia report (Department of Health, 2015). Texts missing sufficient detail were excluded (i.e. study protocols and conference abstracts). We also excluded texts using the “Dementia Champion” term to describe a non-professional role (i.e. volunteers). This meant we excluded papers discussing the UK charity Alzheimer’s Society’s “Dementia Friends Champions”. This term refers to volunteers who have been trained to deliver 60-min sessions developed by the Alzheimer’s Society with basic information about dementia.

**Table 1.** Eligibility Criteria for Review Articles

Exclusion	Inclusion
• Texts produced before 2010 • Use of DC term in non-professional capacity (e.g. volunteers; people with lived experience) • Texts not written in English language • Texts without sufficient information to answer research questions	• National and international texts (written in English) • Texts reporting descriptive/conceptual information • Texts reporting outcome data

Of the eligible texts, we categorised information by service type (i.e. health care, social care, and cross-sector data). We used Microsoft Excel to systematically organise, extract, and synthesise data, using a tabulation process. We extracted descriptive information including: a definition of a DC, who took on the role, and the settings where they worked. Using our ToC approach, we then developed a model of ‘activities/inputs’ to identify the tasks and responsibilities of the role, and the mechanisms of action/supporting systems in place to implement (short-term mechanisms), embed (medium-term mechanisms) and maintain (long-term mechanisms) the role across settings. All mechanisms were catalogued in a main database and grouped into themes. We reported outcome data where available. We did not assess the quality of texts, which is beyond the scope of a narrative review.

### Analysis

We applied narrative content analysis to identify and categorise activities/inputs of DCs across settings. We applied a deductive approach based on a basic ToC framework (see Figure 1), synthesising data to understand the key components as stated above.

**Figure 1.**
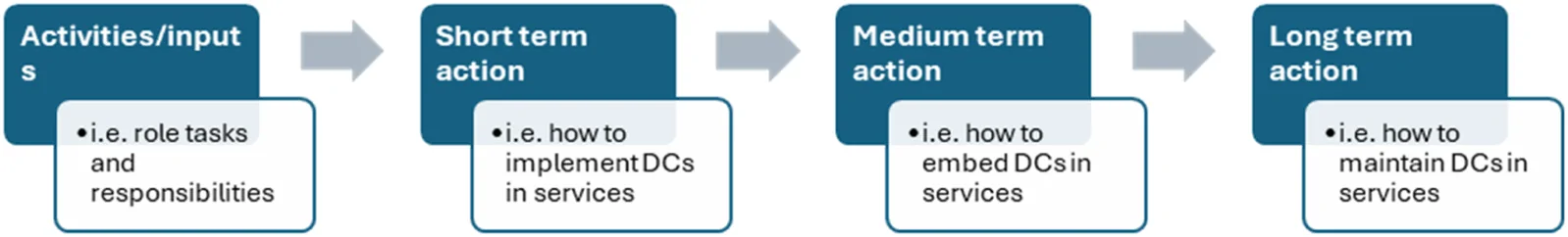
Basic theory of change (ToC) framework

## Findings

### Search Outcomes

We found 27 articles eligible for inclusion. After removing duplicates, database searches produced 609 unique results, with an additional 19 articles identified through hand-searching and citation scanning. We could not access ten full texts. We therefore screened 599 articles against our eligibility criteria, excluding 572 articles (see Figure 2).

**Figure 2.**
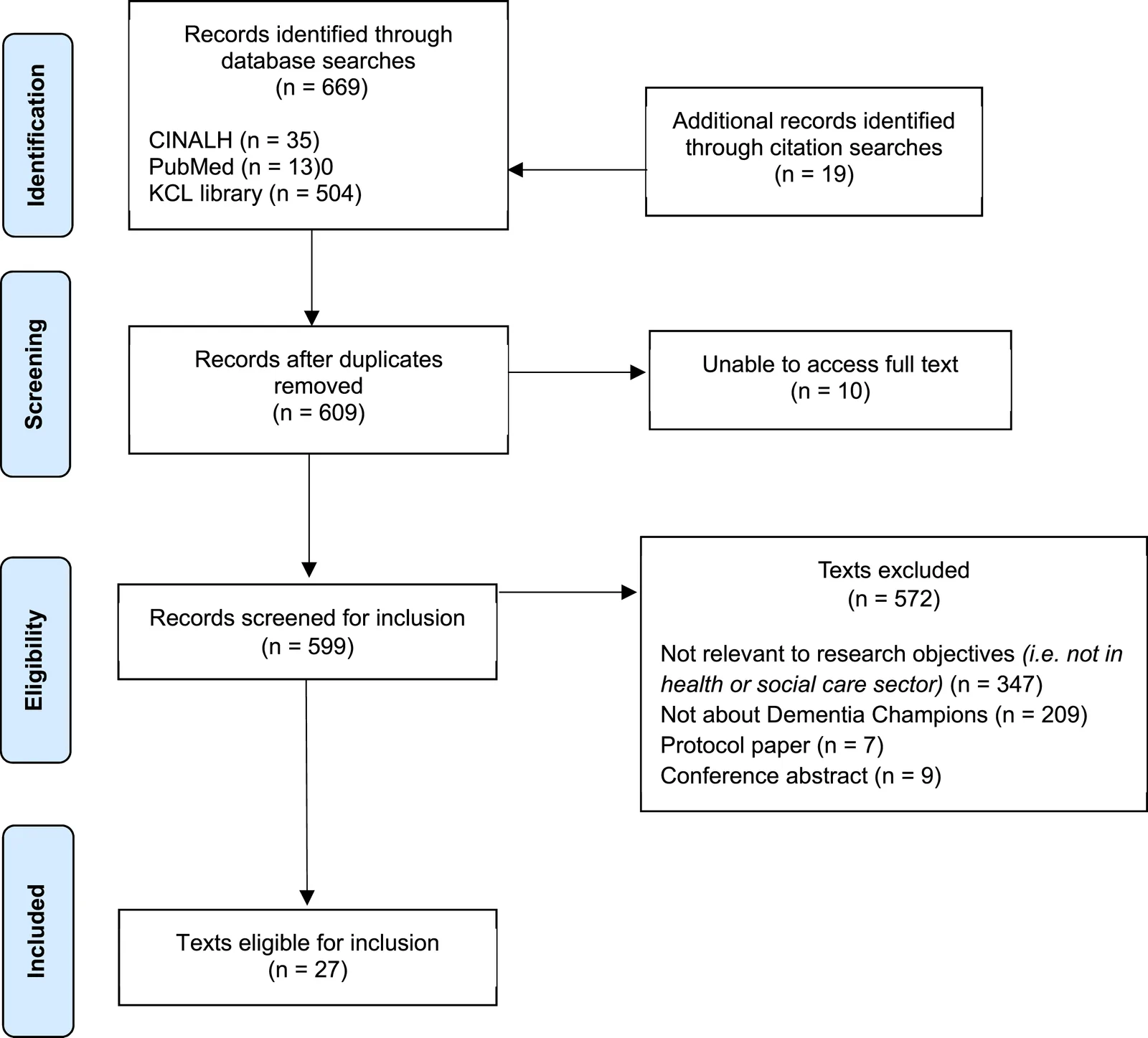
PRISMA-ScR flow diagram illustrating the search, screening, and exclusion process of review articles

Of the 27 articles, 25 (93%) were from the UK, one from Ireland (4%), and one from Australia (4%). Of the UK articles, nine were from Scotland, 14 from England, one from Wales, and one covered Scotland, England and Wales (see Supplementary Material for included article characteristics).

We identified three large programmes of work, accounting for 16 (59%) of the included articles (Table 2). These were: 1) ‘Scotland’s National Dementia Champions programme’ 2) ‘Improving Wellbeing and Health for People with Dementia’ (the ‘WHELD’ programme); 3) ‘Dementia Champion Community of Practice’ (the ‘DEMCoP’ programme).

**Table 2.** Summary of the Three Large Programmes of Work Accounting for 59% of the Included Review Articles

Programmes of work	Summary
Scotland’s national dementia champions programme	Delivered by the University of the West of Scotland in partnership with Alzheimer Scotland which aimed to improve the care of people living with dementia and their families in acute hospital and related settings. As of 2021, more than 1000 people (mostly healthcare professionals) completed the programme, which remains ongoing (Alzheimer Scotland Centre for Policy and Practice, 2023)
Improving wellbeing and health for people with dementia (the ‘WHELD’ programme)	A person-centred psychosocial intervention to improve outcomes for people living with dementia in care homes. The study appointed two DCs per site to deliver the intervention across 69 care homes in England (i.e. Ballard et al., 2018)
Dementia champion community of practice’ (the ‘DEMCoP’ programme)	Developed a virtual community of practice to facilitate knowledge transfer among DCs working across health and social care settings in the East of England

## Descriptive Data

### What Services do DCs Work in?

Where specified, DCs working in healthcare were predominantly in hospital settings (Banks et al., 2014; Beavan, 2014; Crabtree & Mack, 2010; Elliot & Adams, 2012; Ellison et al., 2014; Gkioka et al., 2020; Jack-Waugh, 2023; Jack-Waugh et al., 2011, 2018; Sheaff et al., 2018; Timmons et al., 2016; Trueland, 2012; Wilkinson et al., 2016). Others worked in community mental health services (Brindley, 2011; Mayrhofer et al., 2014, 2016); primary care (Mayrhofer et al., 2014, 2016); and other unspecified outpatient services (Mayrhofer et al., 2014, 2016). In social care settings, DCs worked in day centres (Mayrhofer et al., 2014, 2016); care homes (Ballard et al., 2018, 2020; Elliot & Adams, 2012; Fossey et al., 2019, 2020; Romeo et al., 2019; Sheaff et al., 2018); and other residential facilities (Beer et al., 2011). Three articles did not specify a service, and we found no articles on homecare.

### Who Works as a DC?

In most healthcare settings, DCs were qualified healthcare professionals, such as nurses (Banks et al., 2014; Beavan, 2014; Butler, 2015; Cleland, 2011; Crabtree & Mack, 2010; Ellison et al., 2014; Jack-Waugh, 2023; Jack-Waugh et al., 2018; Trueland, 2012); allied health professionals (Banks et al., 2014; Jack-Waugh, 2023; Jack-Waugh et al., 2018); junior doctors (Wilkinson et al., 2016); and managers (Banks et al., 2014; Ellison et al., 2014; Jack-Waugh et al., 2018). In social care services, DCs were social care staff (unspecified) and social workers (Ballard et al., 2018; Brindley, 2011; Elliot & Adams, 2012; Fossey et al., 2019; Jack-Waugh, 2023; Jack-Waugh et al., 2018; Romeo et al., 2019; Sheaff et al., 2018). In a sample of 514 DCs in one study, 90% were female (Jack-Waugh et al., 2018), but such characteristics were sparsely reported elsewhere.

### How do Staff Become DCs?

Managers sometimes chose staff to become DCs (Ballard et al., 2018; Beer et al., 2011; Elliot & Adams, 2012; Fossey et al., 2019, 2020; Romeo et al., 2019), while others were selected by trained facilitators (Sheaff et al., 2018). Some managers appointed themselves to the DC role (Sheaff et al., 2018). Mayrhofer et al. (2015) found some healthcare providers considered all staff DCs, while others identified key people for the role. In the DEMCoP programme, half the DCs were nominated by others, while half put themselves forward for the role (Mayrhofer et al., 2014).

## Theory of Change (ToC) Framework

### Activities/Inputs: What is the Role of a Dementia Champion?

DCs performed a wide range of tasks across all services and settings. While there was no ‘one-size-fits-all’ approach, there were some key overlapping features. DCs were typically described as leaders and role models with specialised dementia knowledge, who cascaded information and best practice down to other care staff; as agents for change, by improving dementia care for those affected by dementia and the service in which they worked; and above all, as highly knowledgeable and experienced in providing good dementia care.

DCs had overlapping responsibilities that served the service, its care staff, and service users. Service-related tasks included: identifying areas for improvement and influencing development and change (Beavan, 2014; Butler, 2015; Marlowe, 2014); establishing organisational structures and initiatives to improve dementia care (Wilkinson et al., 2016); challenging poor practice (Crabtree & Mack, 2010; Marlowe, 2014); and promoting multi-disciplinary team (MDT) learning and working (Crabtree & Mack, 2010; Marlowe, 2014), i.e. as named contacts across services (Mayrhofer et al., 2015). In UK healthcare, DCs contributed to the development of an NHS Trust-wide dementia strategy (Beavan, 2014), and liaised between settings to improve patient care (Crabtree & Mack, 2010; Mayrhofer et al., 2015). DCs often worked closely with other care staff to improve practice and quality of care.

Staff-related responsibilities involved promoting person-centred care (e.g. by modelling good practice) (Cleland, 2011; Crabtree & Mack, 2010; Elliot & Adams, 2012; Marlowe, 2014; Mayrhofer et al., 2014, 2016; Sheaff et al., 2018; Thomas, 2010; Wilkinson et al., 2016); cascading knowledge (Ballard et al., 2018, 2020; Beavan, 2014; Crabtree & Mack, 2010; Fossey et al., 2019, 2020; Mayrhofer et al., 2014, 2016); providing guidance and support (Beavan, 2014; Cleland, 2011; Crabtree & Mack, 2010; Sheaff et al., 2018; Timmons et al., 2016; Wilkinson et al., 2016), delivering staff training and education (Beavan, 2014; Beer et al., 2011; Elliot & Adams, 2012; Fossey et al., 2019, 2020; Marlowe, 2014; Mayrhofer et al., 2014, 2016; Sheaff et al., 2018); being a named ‘go-to’ person for staff (Mayrhofer et al., 2014, 2015, 2016); improving staff morale (Sheaff et al., 2018; Wilkinson et al., 2016); and continuing the DC programme (Wilkinson et al., 2016).

Service-user and family carer responsibilities included contacting patients and relatives soon after diagnosis (Brindley, 2011); advocating (Crabtree & Mack, 2010; Marlowe, 2014); promoting wellbeing (Crabtree & Mack, 2010); improving and reviewing care plans and assessments (Ballard et al., 2018; Sheaff et al., 2018); and providing education, support, and advice to family members (Brindley, 2011; Cleland, 2011; Crabtree & Mack, 2010; Wilkinson et al., 2016). WHELD DCs received training to write strengths-based, tailored care plans (Ballard et al., 2018).

### Mechanisms of Action

We identified the mechanisms of action required for the DC role to be implemented (short-term mechanisms), embedded (medium-term), and mainlined (long-term) Some mechanisms are cross-cutting and relevant at various time points. The ToC framework is summarised in Table 3.

**Table 3.** Summarised Mechanisms of Action

Theory of change framework Mechanisms of action		
Short-term mechanisms – Implementing DCs	Medium-term mechanisms – Embedding DCs	Long-term mechanisms – Maintaining DCs
1. Organisational commitment - Organisation buy-in - Embed DC role within service culture - Secure funding for ongoing development	1. Role clarity - Establish protected time - Form a cemented identity - Give visibility - Provide written documentation and guidance	1. Organisational and managerial support - Provide regular supervision with management - Support to achieve goals - Upskill, with visible evolution of roles - Ensure ongoing commitment following changes in leadership - Encourage reflective practice - Provide opportunities for ongoing development
2. Role setup - Establish a support network - Provide sufficient training - Set appropriate expectations - Develop an achievable action plan - Engage with specialists - Plan for higher level support - Provide opportunities for reflective practice	2. Working with others - Establish relationships with DCs across services - Adopt a tiered approach to staffing - Ensure respect from colleagues - The ‘go-to’ dementia specialist	2. Achieving expectations - Instil sense of agency to influence change - Adopt measurable goals with efforts to evaluate impact - Provide recognition, encouragement and feedback
3. Recruitment - Select the ‘right’ person - Self-nominated/motivated - Provide equal opportunity to all		3. A wide network of support - Not a lone role - Ensure opportunities for peer support - Create or support DC networking events
4. DC’s motivation - Career step-up/upskilling - Provide certification - Ongoing training opportunities		

### Short-Term – Implementing DCs in Health and Social Care Settings

To implement DCs, the role needs to be firmly rooted into service culture, with evident and planned commitment from the organisation, including sufficient resources and funding, recruiting the ‘right’ person for the role, and a clear plan for role setup.

#### Organisational Commitment

##### Organisational Buy-in

Organisations should be committed to the success of the role from the offset, giving DCs the best opportunity to achieve the intended aims (Jack-Waugh et al., 2011). This includes factoring in protected time for DCs to carry out their responsibilities (Ballard et al., 2020); planning for and providing regular line-management (Ellison et al., 2014; Fossey et al., 2019; Marlowe, 2014); and dedicated training time within working hours. (Trueland, 2012). Organisational bureaucracy can cause challenges across settings, and so it is imperative that employers value DCs’ expertise and their potential impact, encouraging them to create measurable change (Cleland, 2011).

##### Embed Role Within Service Culture

The DC role should be embedded within the service, and not seen as a solo endeavor (Jack-Waugh et al., 2018). An organisational framework with resources to facilitate relationships and provide opportunities for learning is important (Mayrhofer et al., 2015; Sheaff et al., 2018).

##### Secure Funding for Ongoing Development

Long-term investment is needed to include DCs within the workforce, and to ensure ongoing development for DCs to grow within the role (Brindley, 2011).

#### Role Setup

##### Establish a Support Network

Team working and joint sessions with other DCs can help to develop shared goals and build relationships (Sheaff et al., 2018; Wilkinson et al., 2016). This can be achieved by having multiple DCs within a team (Ballard et al., 2018, 2020; Beer et al., 2011). Pre-established mentors and ‘buddy wards’ in hospitals facilitated DC’s wider relationships (Sheaff et al., 2018).

##### Provide Sufficient Training

Training and educational opportunities for DCs was recognised as important (Ballard et al., 2018; Beavan, 2014; Cleland, 2011; Fossey et al., 2019, 2020; Jack-Waugh, 2023; Jack-Waugh et al., 2011, 2018; Mayrhofer et al., 2015, 2016; Romeo et al., 2019; Sheaff et al., 2018; Thomas, 2010; Timmons et al., 2016; Trueland, 2012). In an evaluation of 430 DCs, training positively impacted DCs’ dementia knowledge and attitudes and significantly increased their perceived ability to influence change (Jack-Waugh, 2023; Jack-Waugh et al., 2018). Across studies, training ranged from four months (Ballard et al., 2018, 2020) to over eight months (Jack-Waugh et al., 2018); and used didactic and experiential training principles (Ballard et al., 2018, 2020), face-to-face sessions, distance learning, active participation, reflective writing, and assignments (Jack-Waugh et al., 2018). Training which is made accessible (Fossey et al., 2020); given protected time (Trueland, 2012); tailored around individual support needs and delivered in shorter sessions (Fossey et al., 2020); and delivered in partnership with people living with dementia and their families (Jack-Waugh, 2023) was valued.

##### Other

Other factors which contribute to the role's success in terms of set-up include setting appropriate expectations (Brown et al., 2018) by, for example, having a clear role definition (Mayrhofer et al., 2016); planning for higher level support such as coaching and supervision (Ballard et al., 2018, 2020) or visits from learning facilitators (Crabtree & Mack, 2010); and creating opportunities for DCs to engage in reflective practice (Beer et al., 2011; Fossey et al., 2020; Wilkinson et al., 2016).

#### Recruitment

##### Select The ‘Right’ Person

Important qualities for DCs include motivation, enthusiasm, good communication, and having genuine interest in dementia care (Beer et al., 2011; Crabtree & Mack, 2010; Ellison et al., 2014; Thomas, 2010). Passion alone is not enough (Butler, 2015). The DC title may imply expertise but this needs backing by skills and knowledge (Brown et al., 2018).

##### Self-Nominated/Motivated

The DC role should be advertised to all internal staff equally. Self-nominating DCs may be more motivated and driven (Ballard et al., 2020; Thomas, 2010), and have better expectations from the outset (Thomas, 2010) than those who are nominated by managers or service specialists (Ballard et al., 2018, 2020; Beer et al., 2011; Cleland, 2011; Elliot & Adams, 2012; Fossey et al., 2019, 2020; Sheaff et al., 2018). Providing positive feedback on skills may help motivate staff to become DCs (Fossey et al., 2020), enabling them to see their abilities in a different light.

#### DCs’ Motivation to Take on the Role

The DC title and associated experience on a CV/resume was regarded as beneficial for career development (Trueland, 2012), giving recognition to the upskilled responsibilities (Wilkinson et al., 2016). Across settings, staff wanted a qualification attached to the DC title to recognise their additional knowledge, ability, and responsibilities (Mayrhofer et al., 2014, 2016). Ongoing opportunities such as training and workshops to further dementia education kept DCs motivated (Beer et al., 2011; Wilkinson et al., 2016). Others were motivated through family experience of dementia (Wilkinson et al., 2016).

### Medium Term – Embedding DCs in Health and Social Care Settings

Embedding the DC role requires role clarity to manage expectations and integrate DCs within the workforce, with a support network and plan for how DCs work, whom they work with, and how they are viewed by others.

#### Role Clarity

##### Establish Protected Time

Protected time should be given to the role and training (Ballard et al., 2018, 2020). When the DC role was positioned as an adjunct to work-as-usual, balancing roles led to tasks being completed in personal time (Banks et al., 2014; Ellison et al., 2014).

##### Form a Cemented Identity

A clear role definition is needed to set realistic expectations for DCs and those who draw upon their support (Mayrhofer et al., 2016). DCs need a cemented identity in the workforce to embed within a service, as lack of clarity or associated qualification diminished the role and its value (Mayrhofer et al., 2016). In hospitals, knowing who the DC was and understanding their role encouraged other staff to ask for their support (Wilkinson et al., 2016). To be known as the ‘go-to’ person, DCs need visibility within the service which can be achieved with an identifiable badge or pin (Beer et al., 2011; Mayrhofer et al., 2014); or via information posters in the service (Mayrhofer et al., 2014).

#### Working With Others

DCs benefitted from networking with other DCs (Beavan, 2014) and dementia care staff across services (Jack-Waugh et al., 2018), to share ideas and build relationships. DCs should be involved in continuous development across services, including passing on knowledge to ‘like-minded’ colleagues, and encouraging them to undertake similar learning (Crabtree & Mack, 2010). Where it is not possible or practical to train all staff in dementia care, DCs can cascade their knowledge to improve the overall care experience (Beavan, 2014; Elliot & Adams, 2012; Mayrhofer et al., 2016; Sheaff et al., 2018; Trueland, 2012). Some DCs ran training sessions and delegated responsibilities amongst care home staff, supporting them to deliver the intervention with residents (Fossey et al., 2020), despite some concern from busy care staff (Fossey et al., 2019). Multiple DCs in each service enabled peer support and collaboration (Ballard et al., 2020; Beavan, 2014; Elliot & Adams, 2012). Respect and recognition from colleagues boosted DCs’ confidence and sense of achievement (Fossey et al., 2019).

### Long-Term – Maintaining DCs in Health and Social Care Settings

Maintaining the DC role in practice can be challenging. We identified three important mechanisms of action, including: support from the organisation and management; a wider network of peer support; and the ability for DCs to achieve role expectations.

#### Continued Organisational and Managerial Support

##### Provide Regular Supervision From Management

DCs received regular support with weekly or fortnightly supervision sessions (Ballard et al., 2018, 2020; Fossey et al., 2020). However, support also ended with the WHELD intervention and therefore evidence of sustainability was limited (ibid).

##### Support to Achieve Goals

DCs need organisational support to lead and sustain change (Ellison et al., 2014), as a lack of organisational commitment risked limiting the role’s long-term impact (Mayrhofer et al., 2016). In care homes, DCs attributed personal growth to their role in the intervention, peer support, and strong leadership from managers (Fossey et al., 2019). Junior doctors who became DCs reported feeling better equipped to provide hands on care when they had organisational support (Wilkinson et al., 2016).

##### Upskill, With Visible Role Evolution

In hospitals, the DC title and role itself offered a ‘career boost’, perceived as more beneficial than additional pay (Trueland, 2012). However, increased pay was seen as necessary to reflect the upskilled, complex nature of the role (Mayrhofer et al., 2016). Senior staff were perceived as imperative for developing DCs and cultivating leadership (Sheaff et al., 2018).

##### Ensure Ongoing Commitment Following Changes in Leadership

The above three factors required ongoing organisational commitment following changes in leadership. For example, changes in care home ownership caused uncertainty and frustration around the continuation of the DC role (Fossey et al., 2019).

##### Provide Opportunities for Ongoing Development

DCs need continual opportunities, including training (Elliot & Adams, 2012), to ensure practice (Fossey et al., 2019) and leadership development (Wilkinson et al., 2016), and to prolong motivation (Beer et al., 2011). Consistency in skill-level was needed to visibly improve care for people affected by dementia (Butler, 2015). Conversely, lack of access to associated qualifications or better pay limited the role’s success (Mayrhofer et al., 2016).

#### Achieving Role Expectations

##### Instill Sense of Agency to Influence Change

DCs wanted support to foster service changes in practice and were motivated by having a strong sense of agency (Fossey et al., 2019). Support from senior doctors helped DCs to feel involved in practice development and removed barriers to influence change (Wilkinson et al., 2016), which employers encouraged, but those able to do so were ‘few and far between’ (Butler, 2015). Using a ‘change action plan’ can give DCs measurable objectives to view their progress (Jack-Waugh et al., 2018).

##### Provide Recognition, Encouragement and Feedback

Seeing the positive effect on residents and receiving feedback can help DCs feel recognised (Fossey et al., 2019), as can monthly team awards (Sheaff et al., 2018).

#### A Wide Network of Support

##### Not a Lone Role

The DC role cannot succeed as a lone role, but rather requires a *“weight of numbers… to support change and learning into practice within and across systems”* (Brown et al., 2018). Structured meetings with dementia specialist nurses broadened support networks (Crabtree & Mack, 2010). Multiple DCs in each setting facilitated collaboration through effective peer support, enabling DCs to develop complementary skills (Ballard et al., 2020). Peer support enabled DCs to achieve goals (Fossey et al., 2019), and fostered opportunities for idea sharing and support (Beavan, 2014).

##### DC Networking Events

Networking events can help DCs develop alliances by providing space across services to connect and support each other (Ellison et al., 2014; Jack-Waugh et al., 2011), develop resilience (Ellison et al., 2014), and foster shared identities (Mayrhofer et al., 2015). Across hospitals and care homes, DCs participated in web-based forums, teleconferences, newsletters, monthly team awards, and annual conferences to develop confidence and leadership skills (Sheaff et al., 2018; Fossey et al., 2019).

## Discussion

Through a narrative review, we defined and conceptualised the DC role across health and social care. We developed a ToC framework, identifying the activities/inputs (i.e. tasks and responsibilities), and the short, medium, and long-term mechanisms of action for implementing, embedding, and maintaining the DC role across settings.

Whilst we included articles from across health and social care, no articles were identified from the homecare sector. This sector is an important part of adult social care, with its own intricacies and challenges that will likely impact the mechanisms of action for the DC role in these services. This review forms the first phase of a wider programme of work, which aimed to develop and refine the role of DCs in the homecare sector (King’s College London, 2022; Leverton et al., 2023). The paucity of evidence supports the need for further research to understand how the role can be implemented, embedded, and maintained in homecare.

### Theory of Change (ToC) Framework – Implications for Practice

Activities/inputs (tasks and responsibilities): DCs within and across settings carried out a wide range of tasks, many requiring a high level of skill and leadership capabilities. This suggests DCs are those in more senior-level positions who are experienced in delivering good-practice dementia care. This has implications for DC recruitment (see ‘short-term actions’ below). The degree of variation also highlights poor role definition and consistency across health and social care, which risks the role becoming diluted. Early guidance on older people’s champions identified that few received a job description, which was associated with lack of role clarity and expectations (Department of Health, 2004). Our work highlights that little progression has been made in providing clarity for championing roles 20-years on.

Short-term mechanisms (implementing DCs): Getting the right person in the role is important, but so too is the ability for DCs to be self-selecting to ensure motivation and leadership qualities. Successful implementation requires organisational planning and commitment (i.e. sufficient funds and protected time for training), which we termed ‘organisational buy-in’ to highlight the need for a clear, actionable plan from the outset for role set-up and maintenance.

Medium-term mechanisms (embedding DCs): Role clarity is required to set and manage expectations of, and for, the DC, as well as managers, care staff, service users and families. Organisations need a plan for how DCs will work (i.e. scheduling), who they will work with, and how they will be viewed by others (i.e. visibility). Ensuring protected time is crucial, as DCs can too readily be pulled into regular care duties if services are busy and/or short staffed. Clear visibility of the DC role facilitates respect and recognition.

Long-term mechanisms (maintaining DCs): Organisations need to provide ongoing support, opportunities for upskilling, career development, and recognition. Additional pay is only one way to recognise the upskilled role. DCs must be given the tools and agency to enact change and achieve evident expectations of the role.

This framework has been developed to support DCs in health and care settings, therefore, single mechanisms should not be isolated or considered independently. Doing so may produce unreliable data or undesired effect (Rogers, 2008). Defining and conceptualising the DC role are a start to encouraging consistency in terminology to support care services, networks, and support systems to better implement, embed and maintain these roles within and across services. Consistent terminology would improve role clarity for all stakeholders.

This work revealed a paucity in longitudinal data on the impact of DCs on the service, workforce, and service users, as well as evidence on cost-effectiveness. Where the DC role was actioned as part of an intervention, it was either not sustained after the intervention ended or was not followed-up. For example, the WHELD programme withdrew DC support and assessment when the intervention ended (Ballard et al., 2018). In contrast, Scotland’s National DC programme has expanded to “Dementia Champions International” (University of the West of Scotland, 2019), continuing its success.

### Limitations

As per narrative review methodology, we did not assess article quality. Consequently, the level and quality of information on the DC role varied across articles. Moreover, by focusing solely on the term “Dementia Champion”, articles using different terms for similar specialist roles may have been excluded. However, we did so to identify whether the use of one concise term elicits greater role clarity and homogeneity, rather than opening up our search to include all possible dementia specialist terms. Almost all identified articles (93%) were from the UK, suggesting that the DC term may be less applicable internationally. Countries such as Sweden (Moberg et al., 2018) and Japan (Nakanishi & Nakashima, 2014) where there is greater focus on professionalisation of the dementia workforce, use terms such as ‘home helpers’ to refer to these specialist roles (Japan Academy of Gerontological Nursing, 2012). Additionally, the DC role is practice-specific and so evidence written in non-English languages was not included.

## Conclusion

We conducted a narrative review to conceptualise the role of DCs in health and social care. We developed a ToC framework, establishing DC’s tasks and responsibilities, and identified the mechanisms of action needed to implement, embed, and maintain the role across care settings and services. This work highlighted a lack of consistency in the activities associated with the DC title, and a paucity of longitudinal data on the impact and cost-effectiveness of the role. We found no evidence of DCs in homecare, an important part of the social care sector. We will draw on this framework to facilitate further research to translate knowledge of the DC role across health and social care, to the unique sector of homecare.

## Supplemental Material

Supplemental Material - The Role of Dementia Champions Across Health and Social Care Settings: Identifying Mechanisms of Action Using a Theory of Change Approach Supplemental Material for The Role of Dementia Champions Across Health and Social Care Settings: Identifying Mechanisms of Action Using a Theory of Change Approach by Monica Leverton, Tiffeny James, Kritika Samsi, Christina Newton, Jill Manthorpe in Dementia.
