# Supplementary material for: The Role of Dementia Champions Across Health and Social Care Settings: Identifying Mechanisms of Action Using a Theory of Change Approach
Source: Dementia (London). 2026 Jan 23;25(8):1981–99. doi: 10.1177/14713012251410977 (PMC13683885; doi:10.1177/14713012251410977)
Supplement: Supplementary file 1 [file sj-pdf-1-dem-10.1177_14713012251410977.pdf]

Supplementary material

Review article characteristics (n=27)

Source	Author	Programme	Country	Sector	Service
Academic journal article	Jack-Waugh et al., 2018	Scotland's National Dementia Champion programme	Scotland	Health & Social care	Predominantly hospitals
Academic journal article	Jack-Waugh, 2023			Health & Social care	Predominantly hospitals
Academic journal article	Banks et al., 2014			Health & Social care	Predominantly hospitals
Trade Press	Jack-Waugh et al., 2011			Health care	Hospitals
Report	Ellison et al., 2014			Health & Social care	Predominantly hospitals
Editorial	Brown et al., 2017			Health & Social care	Not specified
Trade Press	Trueland., 2022			Health care	Acute hospitals
Trade Press	Crabtree & Mack, 2010		Scotland	Health care	Hospitals
Academic journal article	Ballard et al., 2018	Well-being and Health for people with Dementia (WHELD) programme	England	Social care	Care homes
Academic journal article	Romeo et al., 2019			Social care	Care homes
Academic journal article	Fossey et al., 2019			Social care	Care homes
Academic journal article	Fossey, Garrod, Guzman, & Testad, 2020			Social care	Care homes
Report	Ballard et al., 2020			Social care	Care homes
Academic journal	Mayrhofer, Goodman, &	Dementia Champion	England	Health & social care	NHS (Acute, Community,

article	Smeeton, 2016	Community of Practice (DEMCoP)			Mental health, Primary care, outpatients, theatre)<br><br>Social care/day services
Report	Mayrhofer, Goodman, & Smeeton, 2014			Health & social care	NHS (Acute, Community, Mental health, Primary care, outpatients, theatre)<br><br>Social care/day services
Discussion piece	Mayrhofer, Goodman, & Holman, 2015			Health & social care	Unspecified (assumed same as above)
Editorial	Marlowe, 2014	-	England	Health care	Not specified
Audit	Timmons, et al., 2016	-	Ireland	Health care	Acute hospital wards
Academic journal article	Beer, et al., 2010	-	Australia	Health and social care	Residential care facilities
Academic journal article	Wilkinson, Coates, Merrick, & Lee., 2015	-	England	Health care	Hospitals
Academic journal article	Sheaff, Sherriff, & Hennessy., 2018	-	England	Health & social care	Hospitals; care homes
Trade Press	Beaven., 2014	-	England	Health care	Acute hospitals
Academic journal article	Elliot, & Adams., 2012	-	England	Health & social care	Acute hospitals; Care homes
Academic journal article	Thomas., 2010	-	England, Scotland, Wales	Health & social care	Care homes
News article	Cleland., 2011	-	England	Health care	Hospitals

News article	Brindley., 2011	-	Wales	Health & social care	Mental health teams
Trade Press	Butler, 2015	-	Scotland	Health & social care	Not specified
